# Effect of a juggling-based physical activity on postural stability, reaction time, and attention focus in older adults: a randomized crossover study

**DOI:** 10.1186/s11556-024-00351-w

**Published:** 2024-05-31

**Authors:** Jakub Malik, Natalia Główka, Wojciech Jelonek, Rafał Stemplewski, Janusz Maciaszek

**Affiliations:** 1 Department of Physical Activity and Health Promotion Science, Poznan University of Physical Education, Królowej Jadwigi 27/39, 61-871 Poznan, Poland; 2 Department of Sports Dietetics, Poznan University of Physical Education, Królowej Jadwigi 27/39, 61-871 Poznan, Poland; 3 Department of Neuromuscular Physiotherapy, Poznan University of Physical Education, Królowej Jadwigi 27/39, 61-871 Poznan, Poland; 4 Department of Digital Technologies in Physical Activity, Poznan University of Physical Education, Królowej Jadwigi 27/39, 61-871 Poznan, Poland

**Keywords:** Physical activity, Dual-task, Cognitive abilities

## Abstract

**Background:**

In the aging society, more attention is paid to the promotion of forms of physical activity that can improve postural stability and cognitive functioning. In this context, the importance of combined exercises, requiring simultaneous physical and cognitive involvement, is emphasized. Juggling seems to be a form of activity that is both cognitively and physically demanding. The purpose of this study was to determine the effect of additional juggling exercise on postural stability and cognitive abilities in healthy, physically active older adults.

**Methods:**

Twenty-six healthy and physically active older adults (70.08±4.40 years old) were included in a randomized crossover study. The addition of juggling three times a week during four weeks was the main intervention (one period), while the control phase included four weeks with no addition of juggling (second period). Measurements of postural stability and cognitive abilities were performed before and after each period. For the purpose of postural stability assessment, a velocity of center of pressure with root mean square, area 95 percentile, medio-lateral and anterior-posterior range of motion were measured. Center of pressure signals were obtained using an AccuGait™ System force plate in three conditions: free standing, dual-task and limits of stability. The Vienna Test System was used for the assessment of selected cognitive abilities. A battery of reaction time tests and Cognitrone test were used for this purpose.

**Results:**

A significant interaction effect of intervention and time was observed in the postural stability dual-task condition in the root mean square of the center of pressure velocity in the advantage of the juggling period (medio-lateral: *F=14.83, p<.01, ƞ*_*p*_^*2*^*=.37;* anterior-posterior: *F=26.30, p<.01, ƞ*_*p*_^*2*^*=.51*). Additionally, moderate effect sizes were observed in the velocity of the center of pressure and variability of simple reaction time measurements, but without statistical significance.

**Conclusions:**

The results of this study indicate that the implementation of juggling activity in everyday life may have positive effects on cognitive abilities and postural stability in healthy, physically active older adults, but the true effect may be low to moderate.

**Trial registration:**

The study was registered retrospectively (30.10.2023) at ClinicalTrials.gov (NCT06108713).

**Supplementary Information:**

The online version contains supplementary material available at 10.1186/s11556-024-00351-w.

## Introduction

A rapidly aging population has been observed nowadays in developed countries [1]. The increasing life expectancy of people, as well as the growing size of the older adult population, is affecting the economy, as well as society as a whole. In view of the mentioned challenges, there is a growing necessity to provide adequate care for older adults [2, 3]. The phenomenon of increasing average age in the global population should, in particular, increase interest in interventions to prevent disease and cognitive decline among older adults. It seems that appropriately targeted physical activity may be a suitable way of healthy aging. Its effects on body composition, cardiovascular diseases [4, 5], diabetes [6] and certain cancers [7, 8] are widely known. In addition, it is increasingly emphasized that physical activity can improve the functionality of aging people [9, 10]. Nevertheless, the activity of older adults does not meet the accepted norm of 150min/week [11]. It is estimated that one in four to five adults does not meet current World Health Organization recommendations in regard to undertaking physical activity [12]. This can accelerate unhealthy aging, which causes both physical and cognitive functional deterioration, further interfering with older adults’ ability to perform daily activities [13]. Therefore, there is a need to organize and recommend attractive and systematic physical activity programs, especially among older adults living in rural areas [14].

Postural stability is of growing interest among the elderly, and they are looking for fall prevention measures to maintain their independence [15, 16]. Among healthy individuals, postural stability relies on somatosensory, vestibular and visual information [17]. Deterioration in one of these functions can create impediments in motor function and the ability to stabilize posture correctly. Additionally, disrupted proprioception significantly decreases postural reactions induced by unexpected stimuli [17, 18]. This phenomenon is connected with the basal ganglia, which is an essential part of the brain involved in maintaining balance [17, 19]. Another factor affecting balance are cognitive abilities [17, 20]. The ability to quick reaction time is important for maintaining balance and avoiding falls during postural challenges or threats [20]. Deterioration of attention and slower reaction time among older adults may be due to changes in the central and peripheral nervous systems [20]. Instability due to cognitive deterioration or age-related changes in the nervous system can lead to a host of problems related to functioning in daily life and health. The greatest risks associated with instability are falls and postural disorders, which are among the great geriatric syndromes and pose a threat to health and life [3]. For example, in 2015, more than 1 million people aged 65 and older in Poland experienced at least one fall. By 2050, this number will almost double [3]. Walking and postural stability is not just a motor task but an activity that engages executive functions and attention [21]. Moreover, exercise has a positive impact on physical functioning, reducing the number of falls and increasing the autonomy of older adults and their quality of life [22]. All these projections show that the care of older adults will be particularly important in the coming years.

In a review on neuronal and cognitive plasticity Greenwood and Parasuraman [23] hypothesized that the interaction between neural and cognitive plasticity is needed for successful cognitive aging. It was stated that whereas neural plasticity is stimulated by experience, cognitive plasticity can change patterns of cognitive behavior [23]. Moreover, the manifestation of cognitive plasticity may depend upon the neural plasticity mechanism [23]. The aging brain has the ability to adapt, among other things, through the promotion of neurogenesis [23, 24] but also through the ability to change in response to the environmental demands of synapses and dendrites [23, 25]. One of the animal studies [26] confirmed that certain plastic changes in the brain can occur in as little as one week. Moreover, synaptogenesis is promoted by novelty, which was confirmed in studies on adult rats in which greater complexity of dendritic trees caused by daily maze exploration tasks was detected [23, 27]. The above conclusions are also confirmed in the case of humans. It has been shown that moderate physical activity, which does not significantly affect a person's cardiorespiratory system but instead engages it cognitively through, among other things, novelty of the task and engagement of attention, has a positive effect on cognition or sensorimotor performance [28–31]. It has also been confirmed that changes in the brain under the influence of this type of activity can take place after just one week of exercise [30, 32].

One such activity that can engage both cognitively and physically and will be a novelty for potential participants is juggling [33, 34]. Classical juggling is a form of activity that requires simultaneous throwing and catching of balls (or other objects like, rings, clubs, scarves) individually with one or both hands in a specific sequence without dropping them [34, 35]. It can be performed in various techniques as column exercises (where each hand throws objects in straight vertical trajectory), parabola exercises (that requires throwing objects from one hand to another) or various (which is a combination of mentioned previously column and parabola exercises) [34]. Although this form of exercise seems demanding, research confirms that older people are fit enough to master juggling exercises [11, 34]. One of the positive aspects of taking up this form of exercise is the growing evidence indicating that juggling improves the well-being of exercisers [34, 36–38]. In addition, our previous research showed that this form of exercise is attractive to older people [34]. Most importantly, the juggling intervention causes an increase in the volume of gray matter [30, 32, 33, 39, 40, 40, 41] and white matter [30, 42, 43] in the human brain and thus shows potential for neuroplasticity. This evidence suggests that engaging in this activity is likely to improve cognitive and executive functions in exercisers, but there is a lack of studies addressing this issue. To the authors’ best knowledge, there are also no studies focusing on the influence of juggling on postural stability.

Therefore, the purpose of this study was to investigate the effect of a juggling intervention on postural stability and selected cognitive abilities in active older adults. We hypothesized that the implementation of a short period of juggling in the everyday life of older adults may improve postural stability, reaction time and attention focus.

## Materials and methods

### Study design

A randomized crossover study design (AB/BA) with two periods (A: implementation of juggling; B: without implementation of juggling) was used. Participants were randomly assigned to the AB and BA groups in a 1:1 ratio. Handedness for homogeneity of the group was examined according to *the Edinburgh Handedness Questionnaire – short form* [44, 45]. Before and after each period, postural stability, reaction time and attention focus were assessed. Physical activity level was tracked using the Polish adaptation of the CHAMPS scale [46].

### Participants

Thirty-seven volunteers aged 65 years or older who responded to a local advertisement were initially enrolled in the study. The inclusion criteria were as follows: physically active people over 65 years old, with no injuries or pathologies involving the upper limb, without neurological issues, and with no significant visual impairments. In turn, individuals with mixed or left-handedness, sedentary lifestyle, any contraindications to physical activity, neurological impairment, daltonism, prior experience in juggling or under the required age were excluded from the study protocol (*n*=7). All participants declared that they took an active part in other organized activities, such as Nordic walking, aqua aerobic or rhythmic gymnastic activities. This information was crucial because among those who had not previously participated in any organized activity, new social factors could affect the measured effects [47]. Participants' physical activity was not restricted during the experiment. However, they were asked not to undertake any new forms of activity for the period of their participation in the project to avoid the motor learning process outside the proposed juggling classes. Dropouts also occurred during the testing and intervention period, including individuals who became seriously ill or missed training units for any reason (*n*=4). Finally, twenty-six participants completed the entire intervention protocol: 21 women and 5 men (age range: 65-75). All procedures were explained and well understood by participants prior to the commencement of the study. All participants signed a written informed consent form and were informed that they could withdraw from participation at any time. The trial was conducted in three waves. First one from September to December 2021, and the next two from March to September 2022.

The study was conducted in accordance with the Declaration of Helsinki 2013 and approved by the Ethics Committee of Poznan University of Medical Sciences (No. 106/21, date: 04.02.2021) and was registered retrospectively at ClinicalTrials.gov (NCT06108713).

### Intervention

**Fig. 1:**
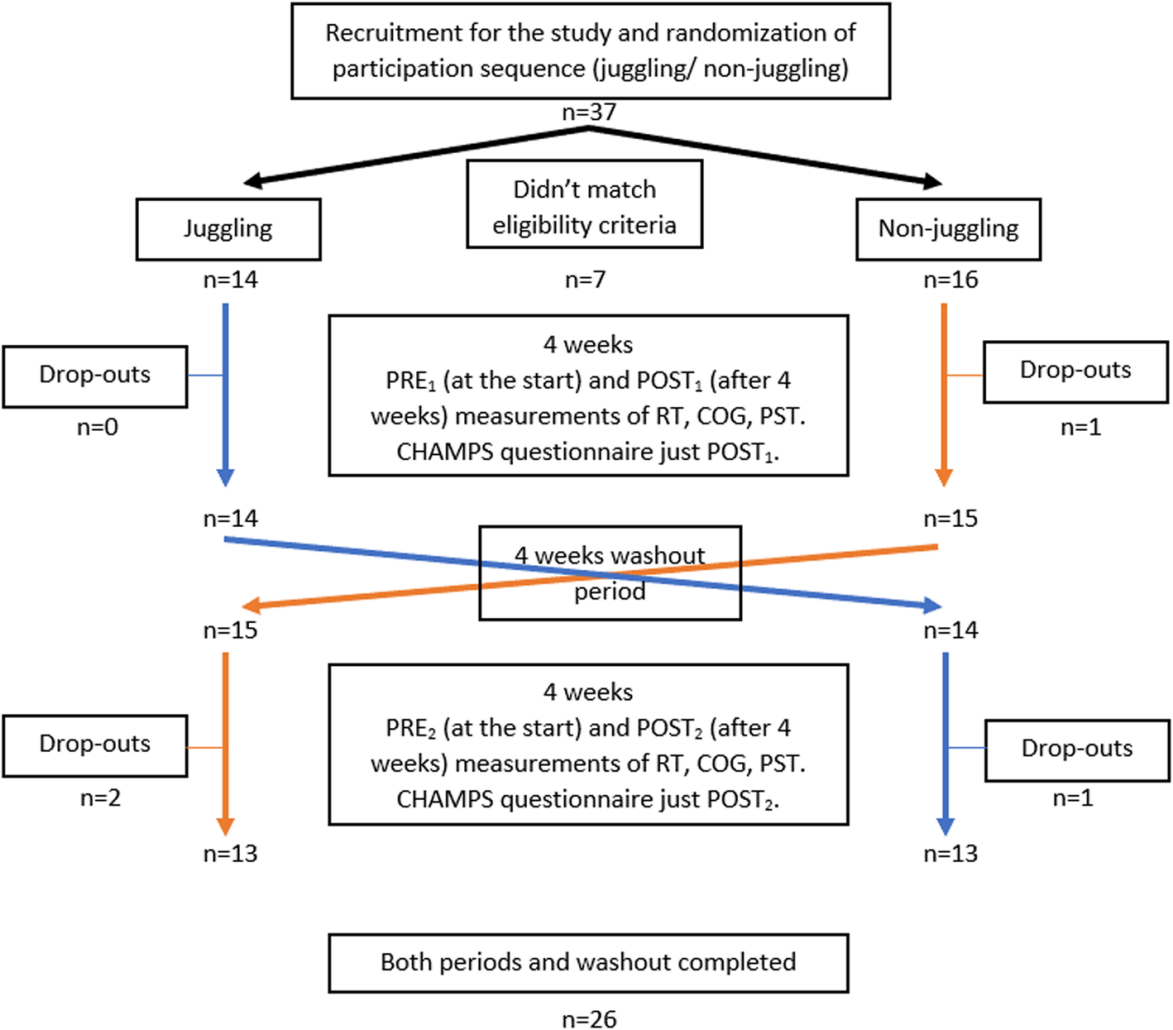
Study design chart; n – number of participants PRE_1_/PRE_2_ – before first/second period; POST_1_/POST_2_ – after first/second period; RT – reaction time tests; COG – attention focus tests; PST – postural stability tests; CHAMPS – physical activity questionnaire

The study took place at Poznan University of Physical Education. Two study periods (juggling period - JP and non-juggling period - NJP) were separated by a four-week washout period. The juggling period lasted 4 weeks and consisted of 12 meetings (3 trainings per week), each lasting 45 minutes [32, 48]. The training was always taught by the same person who is a professional in juggling and has a background in physical education. The first 8 minutes of the training unit was a warm-up with juggling balls and familiarization of the participants with the equipment. The main part of the training unit was dedicated to learning how to juggle three balls cascade. Participants were asked to not juggle outside of the classes. In turn, the last 5 minutes were dedicated to calm down and simple stretching exercises. The entire intervention aimed to execute juggling as a dual-task exercise with the deferral of the traffic automation phase. Each training unit required the completion of various tasks (catching and throwing exercises) using juggling balls. The difficulty level of these tasks increased over time in the JP. To facilitate learning, the balls were in three different colors - green, yellow and red. At a later stage of the intervention, this enabled easy communication of what task was to be performed and which of the several balls held should be used for that task. Additionally, participants completed the course by mastering the basics of the three-ball cascade. During the intervention, participants juggled with 70mm diameter, 90g balls. Due to the complexity of the entire procedure of the intervention, it was described separately in our previous study [34]. Due to the pandemic risk (COVID-19), the groups were intentionally small. A maximum of 5 people participated in one training session at one time so that adequate spacing could be maintained between other exercisers. The NJP was characterized by a lack of additional activity in the form of juggling. The whole study design is described in Fig. 1.

### Measurements

#### Postural stability tests (PST)

The postural stability of the participants was measured using an AccuGait™ System force plate (AMTI PJB-101 model, AMTI, Watertown, MA) with Balance Trainer software and a sampling frequency of 100 Hz. All obtained data were filtered out with a cutoff level of 10Hz [49]. Each participant took part in five trials in a random order. Two trials involved free standing without an additional task (PST_C_), the next two involved free standing with an additional cognitive task (PST_E_), and one trial involved stability limits (PST_LOS_). The PST_C_ mapped the free standing in one minute. Each participant was instructed with the words “*Please stand freely, keep your arms along your torso, eyes open, gaze ahead”*. In the case of the PST_E_ trials, an additional task while standing was to listen to a set of digits and count the occurrence of even or odd numbers (randomized). The numbers were prerecorded sound samples by one of the researchers, played from speakers 1m away from the participant’s backs. The participants heard 30 digits during each PST_E_ trial, which appeared every two seconds. After the trial, the participant reported the result. A mistake of more than 2 was interpreted as poor focus on the task, requiring a retest with a new sound sample. The instructions for each PST_E_ trial were the same, and the exception was the sentence added at the end: *“Count even/odd digits.”*. PST_LOS_ was performed with the assurance of the researcher. Any precipitation beyond the limit of stability caused the test to be repeated. The test participant’s task was to lean forward and backwards and lean to the left and to the right, as much as possible.

Each of the trials lasted 60 seconds. Between each trial, the test participant had a two-minute break in a sitting position. After instructions preceding the test, the researcher waited two seconds before communicating *“start”* and another two seconds before turning on the measurement. Measurements in each period began with familiarization with the PST_C_, PST_E_ and PST_LOS_ trials.

During postural stability trials, center of pressure (COP) displacements were monitored. Based on the COP signal, the following parameters were calculated: velocity (Vcop), area 95th percentile (Area95), medio-lateral range of sway (RangeX), anterior-posterior range of sway (RangeY), medio-lateral root mean square velocity (RMS_VX_) and anterior-posterior root mean square velocity (RMS_VY_) of COP [50]. The reliability of intersession Vcop measurement in older adults is equal to r=.76, for PST_LOS_ variables varies from r=.67 to r=.85 [51].

#### Simple reaction time (SRT) and Choice reaction time (CRT)

Reaction time tests were conducted with the usage of the Vienna Test System (VTS) (SCHUHFRIED GmbH, Austria; Polish distribution - COGNIFIC) with a special panel device. By using the resting and reaction key on the device, it was possible to split the variables into reaction time (RT) and motor time (MT), with both variables measured in milliseconds. The S1 test form was used to assess single-choice responses with a simple visual cue (yellow light on the monitor screen). The S4 test form was used to assess choice reaction time (go/no go) responses with more stimuli (yellow light, red light, yellow and red light simultaneously or simple sound – in various combinations). For S4, the only correct response was in the simultaneous presence of yellow and red light. It was important to use just one finger of the dominant hand to respond in both tests. Data were also collected on the variability of the individual variables (VSRT; VCRT; VMT). The term “reaction time” means the time between the presentation of the stimulus and the occurrence of the response (raising the finger from the resting button). “Motor time” is understood as the time between raising a finger from the rest button and pressing the reaction time button. To standardize the measurements, each approach was preceded by a practice test, after which the participant performed a four-minute main test. Additionally, decision time (DT) variables were calculated from SRT and CRT data using the mental chronometric method (CRT-SRT=DT) [52]. Reliabilities of RT variables vary between r=.83 and r=.98 in the norm sample. MT reliability varied between r=.84 and r=.95 (SCHUHFRIED GmbH).

#### Attention and concentration measurement

The assessment of the attention and concentration of participants was possible through the comparison of figures concerning their congruence in the Cognitrone test form of VTS software. Cognitrone is based on Reulecke’s theoretical model [53], where concentration as a state is described by three variables: (a) energy, (b) function, and (c) precision. We used the S10 form of this test. The task was to answer whether the model displayed below was present in the set of figures at the top of the monitor screen. Participants used both hands. Left hand over the red button (to reject), the right hand over the green button (to accept). Every reaction was recorded by milliseconds and correctness (COG_CR_ – average time of correctly rejected; COG_CA_ – average time of correctly accepted). Skipping a task, going back to a previous or correcting task, was not possible. The correct answer (COG_C_) was to reject correctly (red button if the pattern did not occur in the set of figures) or to accept correctly (green button if the pattern occurred in the set of figures). In addition, the time it took to complete the entire test was also measured (COG_T_). For standardization purposes, each approach was preceded by a practice test, after which the participants performed the main test. The reliability was over r=.95 (SCHUHFRIED GmbH).

#### CHAMPS questionnaire

For the purpose of the study, we used a Polish adaptation of the CHAMPS scale [46]. This questionnaire with 41 questions for assessing the frequency and duration of various physical activities of older adults was used [54, 55]. It is one of the most valid and reliable questionnaires for this purpose [46, 56, 57]. The one-week test-retest reliability of the Polish adaptation was .79 to .85. The questions covered the last four weeks of the participant’s activity and allowed the estimation of caloric expenditure and the frequency of physical activity per week [55]. Study participants were asked to complete a questionnaire at the end of the NJP and JP.

### Outcomes

The primary outcomes of the study were postural stability variables (Vcop including RMS data, RangeX, RangeY and Area95) and selected cognitive abilities (SRT, CRT, MT including variability of these data, DT, COG_CR_, COG_CA_, COG_C_ and COG_T_). Physical activity results from the CHAMP scale were characterized as secondary results.

### Sample size and randomization

For the purpose of the crossover research design, the a priori F-test repeated measure analysis of variance (ANOVA RM), within-between interactions in G*Power software (version 3.1.9.6, Germany) was used [58]. Based on data where Multi System Physical Exercise was used to develop reaction time in older adults (Cohen’s f=.715, what equals of high effect size η_p_^2^=.34 [59]), we chose borderline result of high effect size η_p_^2^=.138, which gives an effect size f(U)=.40 to estimate minimum sample size. According “as in SPSS” option [60], minimal power .80 and alpha error of probability .05, the sample size of 26 participants would be adequate to minimize Type I and Type II errors. Additionally, in two studies with parallel design, significant changes in brain structure after juggling in 12 healthy young participants who underwent the intervention [39] and also in 25 healthy older participants of the intervention group were detected [33]. These results seem to confirm that the estimated minimum sample size should be adequate to detect whether and to what extent juggling can affect functional changes.

Participants enrolled for the study were randomly assigned to one of two groups with the use of randomization with the NumPy library in Python (each participant was assigned a number of 1 to 37 according to the order in which they signed up to participate). These numbers were then input into the array using the *array()* function. Then, their order was randomly generated using the command *Shuffle()*. The first 18 numbers that represented participants from the generated list were involved in the AB sequence of the study, while the remaining numbers were involved in the BA sequence.

### Data analysis

All data analyses were performed using STATISTICA software (version 13.3.0, TIBCO Software Inc., Palo Alto, CA, USA; 2017). Data were analyzed using two-way ANOVA RM to study the interaction between interventions (JP, NJP – as “TR”) and time (PRE, POST – as “PP”) for tests of cognitive function and balance. Differences between the physical activity levels for the two periods were determined using the paired t test (normal distribution) or Wilcoxon test (abnormal distribution). The assumed alpha level was .05. The effect size (ES) was determined from Cohen’s with a 95% confidence interval (95%CI_ES_). Differences are presented as the mean difference (MD) and 95% confidence interval of this value (95%CI_MD_).

## Results

All participants (age=70.08 years old) were right-handed. Detailed characteristics of the participants who finished the whole protocol are described in Table 1.

**Table 1:** Characteristics of participants included into analyses

Variable	All(*n*=26) Mean (SD)	Women(*n*=21) Mean (SD)	Men(*n*=5) Mean (SD)
**Age [years]**	70.08 (4.40)	70.81 (5.10)	69.00 (3.81)
**Body height [cm]**	161.23 (7.89)	158.52 (5.01)	172.60 (8.62)
**Lateralization**^**a**^	92.31 (12.62)	92.86 (12.22)	90.00 (16.30)
**Body mass [kg]**	67.77 (13.44)	63.95 (9.74)	83.80 (16.05)
**BMI [kg/m**^**2**^**]**	25.95 (3.86)	25.49 (3.95)	27.90 (3.01)

^a^Values from 100 to 61 – right-handedness, -60 to 60 mixed handedness, -61 to -100 left-handedness

### Primary outcomes

**Fig. 2:**
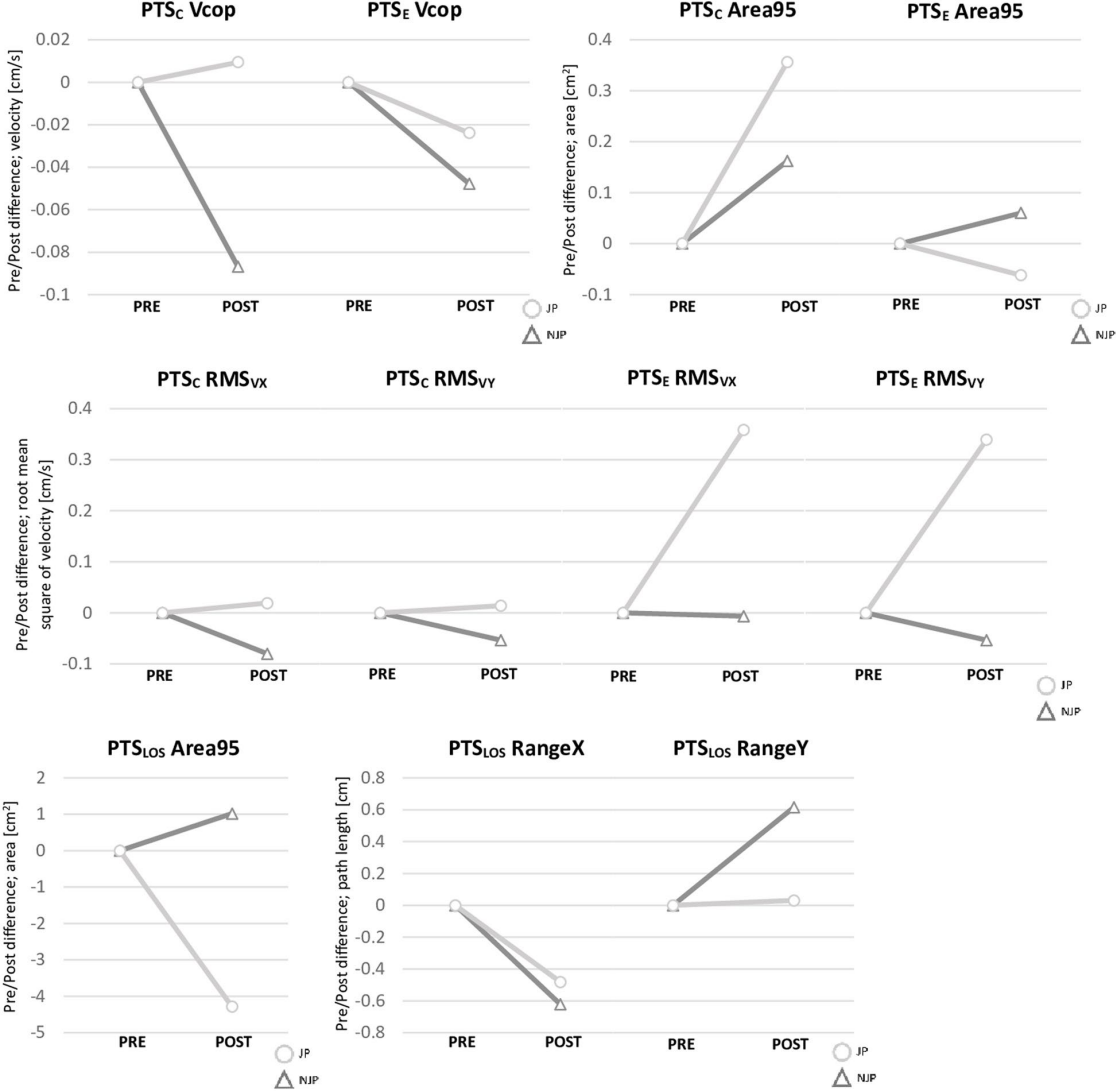
PRE/POST differences in postural stability tests. PST_C_ – postural stability tests without additional task; PST_E_ – postural stability test with additional task (counting); PST_LOS_ – limits of stability test, Vcop – velocity of center of pressure, RMS_VX_ – medio-lateral root mean square of velocity; RMS_VY_ – anterior-posterior root mean square of velocity; Area95 – area 95 percentile, RangeX – medio-lateral range of sway, RangeY – anterior-posterior range of sway, JP – juggling period, NJP – non juggling period

PST_C_ was characterized by the lowest values of Vcop, RangeX, RangeY and RMS_VX_ for POST JP. Additionally, changes in the main PST variables in both conditions from PRE to POST measurements showed an advantage of JP (Fig. 2). For PST_E,_ the lowest values in POST JP were observed for Vcop. However, statistical significance of the interaction of “TR”x“PP” was observed for RMS_VX_ and RMS_VY_ in PST_E_. Bonferroni post hoc analysis of RMS_VX_ showed statistically significantly higher values for POST NJP versus the other time points (PRE JP – POST NJP: *MD=-.37; 95%CI*_*MD*_*=[-.56;=-.18]*; POST JP – POST NJP: *MD=-.38; 95%CI*_*MD*_*=[-.57;-.18]*; PRE NJP – POST NJP: *MD=-.36; 95%CI*_*MD*_*=[-.55;-.17]*). The RMS_VY_ variable was significantly lower in PRE NJP than in other time points of this study (PRE JP – PRE NJP: *MD=.36; 95%CI*_*MD*_*=[.21;.52]*; POST JP – PRE NJP: *MD=.31; 95%CI*_*MD*_*=[.15;.46]*; PRE NJP – POST NJP: *MD=-.34; 95%CI*_*MD*_*=[-.49;-.18]*). Additionally, a statistically significant main effect of “TR” was observed for RangeY JP – NJP: *MD=-.19; 95%CI*_*MD*_*=[-.37;-.02];* PRE JP – PRE NJP: *MD=-.07; 95%CI*_*MD*_*=[-.42;-.29];* POST JP – POST NJP: *MD=-.32; 95%CI*_*MD*_*=[-.68;-.03]*) in the PST_C_ condition. There were no statistically significant differences for any of the PST_LOS_ variables. All data of the three conditions in balance testing are described in Table 2. An additional file shows more detailed post hoc results [see Additional file 1].

**Table 2:** Values of three condition PST measurements (mean, SD, F, *p*-value, effect size and 95%CI_ES_) obtained by participants (*n*=26) during the crossover study

**Variable**	**JP**		**NJP**		**“TR”x”PP”**		**„TR”**		**„PP”**	
	**PRE** **Mean(SD)**	**POST** **Mean(SD)**	**PRE** **Mean(SD)**	**POST** **Mean(SD)**	**F (*p-value*)**	**ƞ**_**p**_^**2**^** [95%CI**_**ES**_**]**	**F (*p-value*)**	**ƞ**_**p**_^**2**^** [95%CI**_**ES**_**]**	**F (*p-value*)**	**ƞ**_**p**_^**2**^** [95%CI**_**ES**_**]**
**PST**_**C**_										
Vcop [cm/s]	1.17(.33)	1.09(.28)	1.17(.27)	1.18(.28)	3.38(*.08*)	.12[.00;.35]	1.47(*.24*)	.06[.00;.27]	1.09(*.31*)	.04[.00;.25]
RangeX [cm]	1.94(.69)	1.87(.69)	2.00(.56)	1.98(.75)	.09(*.77*)	<.01[.00;.15]	.78(*.39*)	.03[.00;.23]	.21(*.65*)	<.01[.00;.17]
RangeY [cm]	2.65(.58)	2.54(.72)	2.71(.67)	2.86(.61)	2.16(*.15*)	.08[.00;.31]	5.24(***.03***)	.17[.00;.41]	.04(*.84*)	<.01[.00;.12]
Area95 [cm^2^]	2.82(1.22)	2.98(1.83)	3.18(1.56)	3.53(2.14)	.40(*.53*)	.02[.00;.20]	3.55(*.07*)	.12[.00;.36]	1.71(*.20*)	.06[.00;.29]
RMS_VX_[cm/s]	.84(.32)	.76(.29)	.84(.25)	.86(.27)	2.71(*.11*)	.03[.00;.33]	1.53(*.23*)	.06[.00;.28]	.53(*.47*)	.02[.00;.21]
RMS_VY_[cm/s]	1.16(.36)	1.11(.31)	1.16(.30)	1.17(.28)	1.46(*.24*)	.06[.00;.27]	.58(*.45*)	.02[.00;.22]	.35(*.56*)	.01[.00;.19]
**PST**_**E**_										
Vcop [cm/s]	1.13(.29)	1.08(.27)	1.14(.27)	1.12(.25)	.09(*.76*)	<.01[.00;.15]	.27(*.61*)	.01[.00;.18]	.81(*.38*)	.03[.00;.23]
RangeX [cm]	1.77(.51)	1.83(.68)	1.88(.60)	1.93(.54)	.01(*.90*)	<.01[.00;.08]	1.94(*.18*)	.07[.00;.30]	.39(*.54*)	.02[.00;.20]
RangeY [cm]	2.50(.57)	2.50(.59)	2.60(.77)	2.47(.62)	.02(*.90*)	<.01[.00;.10]	.04(*.85*)	<.01[.00;.12]	1.06(*.31*)	.04[.00;.25]
Area95 [cm^2^]	2.63(1.25)	2.69(1.30)	3.04(1.49)	2.98(1.46)	.15(*.70*)	<.01[.00;.34]	2.78(*.11*)	.10[.00;.33]	.00(*1.00*)	.00[.00;.00]
RMS_VX_[cm/s]	.78(.30)	.77(.31)	.79(.25)	1.15(.30)	14.83(***<.001***)	.37[.09;.58]	14.81(***<.001***)	.37[.09;.58]	11.55(***<.001***)	.32[.05;.53]
RMS_VY_[cm/s]	1.15(.29)	1.10(.25)	.79(.20)	1.13(.30)	26.30(***<.001***)	.51[.21;.68]	20.75(***<.001***)	.45[.15;.63]	6.09(***.02***)	.20[<.01;.43]
**PST**_**LOS**_										
RangeX [cm]	17.35(4.37)	16.73(5.44)	17.15(4.24)	16.67(5.16)	.01(*.93*)	<.01[.00;.08]	.02(*.88*)	<.01[.00;.10]	.55(*.46*)	.02[.00;.21]
RangeY [cm]	14.86(2.97)	15.47(3.34)	15.64(2.08)	15.68(2.47)	.40(*.54*)	.02[.00;.20]	1.33(*.26*)	.05[.00;.27]	.58(*.45*)	.02[.00;.22]
Area95 [cm^2^]	166.47(71.09)	167.48(84.29)	185.47(83.88)	181.18(91.88)	.04(*.84*)	<.01[.00;.12]	1.74(*.20*)	.07[.00;.29]	.02(*.89*)	<.01[.00;.10]

“*TR*” main effect, intervention (juggling/ non-juggling), “*PP*” main effect: time (PRE/POST), *JP* juggling period, *NJP* non-juggling period, *PRE* – before period, *POST* after period, *F* F statistic of ANOVA RM, *ƞ*_p_^2^ – effect size, partial eta square; 95%CI_ES_ – 95% confidence interval for effect size; PST_C_ – standing freely without an additional task, PST_E_ – standing freely with an additional task; PST_LOS_ – limits of stability test, Vcop – velocity of center of pressure, Area95 – area 95 percentile, RangeX – medio-lateral range of sway; RangeY – anterior-posterior range of sway; RMS_VX_ – medio-lateral root mean square of velocity, RMS_VY_ – anterior-posterior root mean square of velocity

**Fig. 3:**
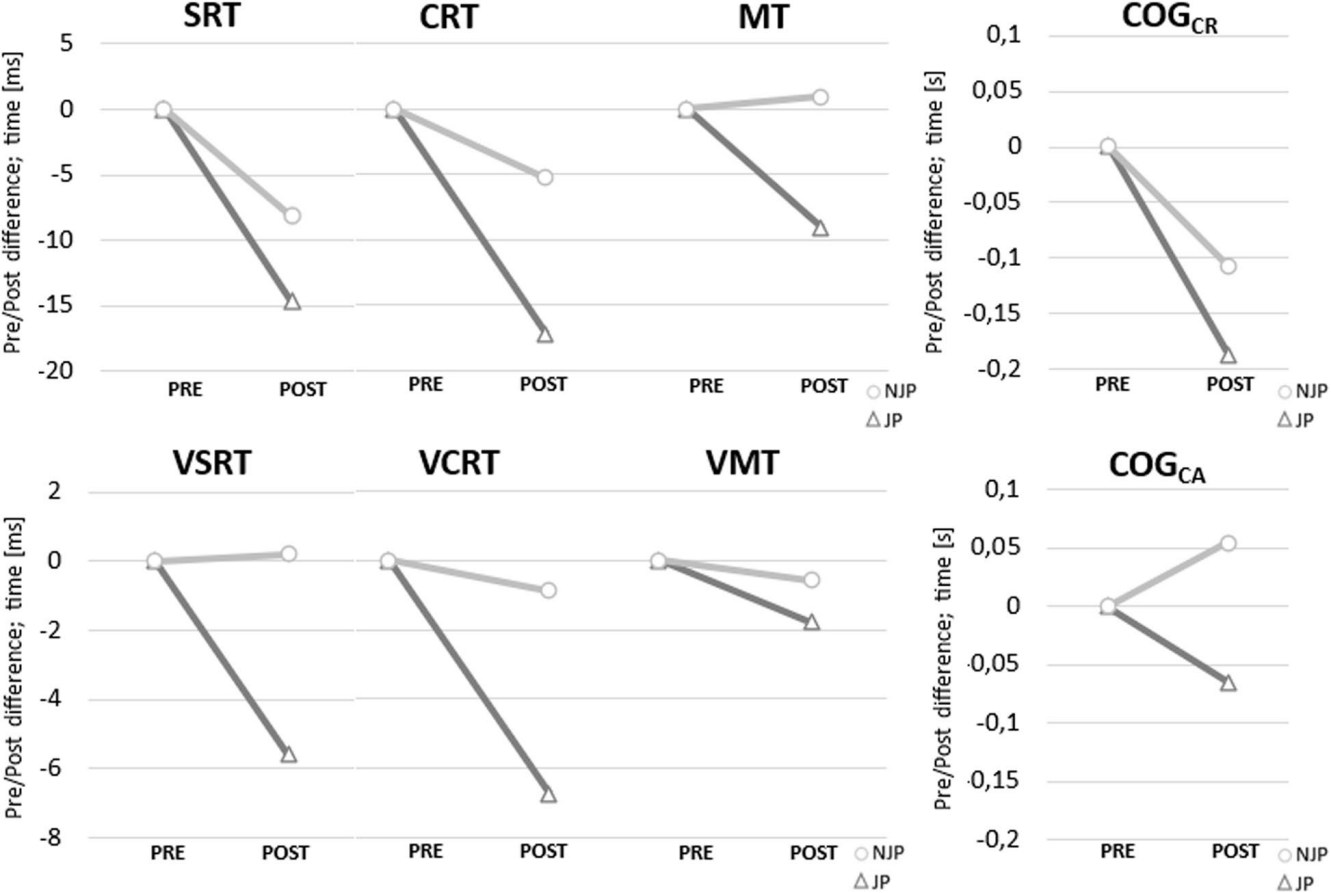
PRE/POST differences in cognitive function assessment. SRT – simple reaction time, CRT – complex reaction time, MT – motor time, VSRT – variability of simple reaction time, VCRT – variability of complex reaction time, VMT – variability of motor time, COG_CR_ - average time of correct rejections in the Cognitrone test, COG_AR_ – average time of correct acceptance in the Cognitrone test, JP – juggling period, NJP – non-juggling period

Participants were characterized by shorter response time between POST JP and the other time points, especially for SRT, VSRT, CRT, VCRT, DT and COG_T_. Additionally, changes in selected cognitive abilities in both conditions from PRE to POST measurements showed an advantage of JP (Fig. 3). Statistical significance was obtained for the main effect of “PP” in variables: SRT and COG_C_. Bonferroni post hoc analysis of SRT showed significantly worse reaction time for PRE compared to POST timepoint (PRE – POST: MD=11.42; 95%CI_MD_=[1.55;21.30]), but there were not significant changes in specific periods as juggling (PRE JP – POST JP: MD=14.69; 95%CI_MD_=[-1.24;23.63]) or control (PRE NJP – POST NJP: MD=8.15; 95%CI_MD_=[-7.78;24.09]) despite a larger change in sequence with the intervention. Bonferroni post hoc analysis for COG_C_ showed significantly less correct answers in Cognitrone test for POST compared to PRE timepoint (PRE – POST: MD=-.81; 95%CI_MD_=[-1.52;-.10]), but without significance in specific periods as juggling (PRE JP – POST JP: -.62; 95%CI_MD_=[-2.57;1.34]) or control (PRE NJP – POST NJP: MD=-1.00; 95%CI_MD_=[-2.96;.96]). . Other cognitive variables were not characterized by any significant changes (*p*>.05). All cognitive data obtained in the study are described in Table 3. An additional file shows more detailed post hoc results [see Additional file 1].

**Table 3:** Reaction time and attention values (mean, SD, F, *p*-value, effect size and 95%CI_ES_) obtained by participants (*n*=26) during the crossover study

**Variable**	**JP**		**NJP**		**“TR”x”PP”**		**„TR”**		**„PP”**	
	**PRE** Mean(SD)	**POST** Mean(SD)	**PRE** Mean(SD)	**POST** Mean(SD)	**F (*p-value*)**	**ƞ**_**p**_^**2**^** [95%CI**_**ES**_**]**	**F (*p-value*)**	**ƞ**_**p**_^**2**^** [95%CI**_**ES**_**]**	**F (*p-value*)**	**ƞ**_**p**_^**2**^** [95%CI**_**ES**_**]**
**Reaction Time test**										
SRT[ms]	307.38(42.97)	292.69(41.98)	303.12(49.89)	294.96(47.65)	.69(*.41*)	.03[.00;.22]	.03(*.87*)	<.01[.00;.11]	5.68(***.03***)	.19[.00;.42]
VSRT[ms]	44.96(17.34)	39.35(11.19)	40.62(14.58)	40.81(15.76)	1.97(*.17*)	.07[.00;.30]	.25(*.62*)	<.01[.00;.18]	1.75(*.20*)	.07[.00;.29]
CRT[ms]	480.35{57.63)	463.12(55.68)	477.81(62.91)	472.54(57.71)	.78(*.39*)	.03[.00;.23]	.30(*.59*)	.01[.00;.19]	3.22(*.08*)	.11[.00;.35]
VCRT[ms]	73.77(18.75)	67.04(20.19)	72.00(20.83)	71.12(19.41)	.92(*.35*)	.04[.00;.24]	.17(*.68*)	<.01[.00;.17]	1.33(*.26*)	.05[.00;.27]
MT[ms]	262.96(73.85)	253.92(71.97)	247.71(64.28)	248.65(81.35)	.68(*.42*)	.03[.00;.22]	3.03(*.09*)	.11[.00;.34]	.43(*.52*)	.02[.00;.20]
VMR[ms]	35.42(8.30)	33.65(11.15)	33.67(9.95)	33.10(13.68)	.20(*.66*)	<.01[.00;.17]	.60(*.44*)	.02[.00;.22]	.52(*.48*)	.02[.00;.21]
DT[ms]	172.96(40.87)	170.42(48.82)	174.69(45.28)	177.58(36.69)	.23(.*64*)	<.01[.00;.18]	.36(*.55*)	.01[.00;.19]	.00(*.97*)	<.01[.00;<.01]
**Cognitrone test**										
COG_CR_[s]	3.23(.94)	3.04(.66)	3.18(.78)	3.07(.73)	.24(*.63*)	<.01[.00;.18]	.04(*.85*)	<.01[.00;.12]	2.97(*.10*)	.11[.00;.34]
COG_CA_[s]	2.52(.61)	2.45(.59)	2.45(.51)	2.50(.58)	1.57(*.22*)	.06[.00;.28]	.02(*.89*)	<.01[.00;.10]	.01(*.91*)	<.01[.00;.08]
COG_C_[n]	54.54(3.42)	55.15(4.20)	54.69(3.67)	55.69(3.87)	.16(*.69*)	<.01[.00;.16]	.55(*.46*)	.02[.00;.21]	5.47(***.03***)	.18[.00;.42]
COG_T_[s]	176.81(46.06)	168.31(35.19)	174.81(40.66)	171.15(35.07)	.55(*.47*)	.02[.00;.21]	.01(*.92*)	<.01[.00;.08]	2.06(*.16*)	.08[.00;.30]

“TR” – main effect: intervention (juggling/ non-juggling); “PP” – main effect: time (PRE/POST), *JP* juggling period, *NJP *non-juggling period, *PRE *before period, POST – after period, *F*   F statistic of ANOVA RM, *ƞ*_p_^2^– effect size, partial eta square, 95%CI_ES_ – 95% confidence interval for effect size, Vienna Test System - Reaction time test: *SRT * simple reaction time, *VSRT * variability of simple reaction time, *CRT*  choice reaction time, *VCRT * variability of choice reaction time, *MT*   motor time, *VMT*   variability of motor time, *DT*  decision time, Vienna Test System - Cognitrone test: COG_CR_ – average time of correct rejections, COG_CA_ – average time of correct acceptance, COG_C_ – number of correct answers, COG_T_ – duration of test, ms – milliseconds, s – seconds, n – number of correct answers

### Secondary outcomes

Participants reported statistically significant higher frequency of total physical activity (AAF) during NJP than JP (*p*=.03) and also in moderate physical activity (MAF; *p*<.05). Nevertheless, data on both: estimated caloric expenditure/week for all activities (ECE) and estimated caloric expenditure/week for moderate only activities (MECE) showed no difference at a statistically significant level (respectively *p*=.06, *p*=.15). Table 4 shows the detailed results of the CHAMPS questionnaire variables.

**Table 4:** Self-reported level of physical activity after JP and NJP (CHAMPS questionnaire)

**Measure**	**JP** Mean (SD)	**NJP** Mean (SD)	**t or Z** (***p*-value)**	**ES** **[95%CI**_**ES**_**]**	**MD *(JP-NJP)*** **[95%CI**_**MD**_**]**
**AAF/week**	28.23(10.31)	31.15(9.92)	-2.34 (.03)*	d=.29 [-.26;.84]	-2.92 [-5.49;-.35]
**MAF/week**	8.42(5.04)	10.00(5.66)	-2.09 (<.05)*	d=.29 [-.25;84]	-1.58 [-3.13;-.03]
**ECE/week**	5747.15(4123.58)	7285.81(3997.35)	1.87 (.06)	d=.37 [-.17;.93]	-1538.67 [-3146.47;69.13]
**MECE/week**	5155.30(3729.85)	6318.70(3597.24)	1.44 (.15)	d=.29 [-.23;.87]	-1163.40 [-2706.02;379.23]

*JP* juggling period, *NJP* non-juggling period, *t or Z* t value for paired t test, or Z value of Wilcoxon test, *ES* effect size, *MD* mean difference, 95%CI_ES_/95%CI_MD_ – 95% of confidence interval for effect size or mean difference, *AAF* all activities frequency, *MAF* moderate activities frequency, *ECE* estimated caloric expenditure/week for all activities, *MECE* estimated caloric expenditure/week for moderate only activities

^*^p<.05

## Discussion

The purpose of this study was to determine the effect of additional juggling exercise on postural stability and cognitive abilities in healthy, physically active older adults. Twenty-one women and five men over the age of 65 participated in the study. This gender difference in the number of participants may have been due the fact that the women more often than men take part in individual activities rather than team activities [61, 62]. Additionally the recruitment process, relied on a request for participation, in a Polish population, where there is a preponderance of women gender among adults over 60 years of age [63] may also contributed to this disparity.

Classical juggling is a kind of activity in which exercisers need to use only the upper limbs of their body. However, if juggling is performed in a standing position or walking, it requires from participants proper postural control. It is a core element within the coordination of most skills. During juggling exercises, the whole attention is directed on the tossed utensils, not on the lower limbs or the body posture [64, 65]. In our study, a statistically significant interaction effect of intervention and time was observed for medio-lateral and anterior-posterior root mean square of COP velocity and during postural stability test with additional counting, with a definite deterioration of medio-lateral root mean square of velocity during the period without intervention. The lowest and statistically significant result of anterior-posterior root mean square velocity was observed at the start of the non-juggling period, since no carryover effects were observed during the study. Deterioration, however, was observed for the non-juggling period, whereas for the juggling period, it remained steady at a level similar to the POST non-juggling period. These results allow us to conclude that the addition of juggling even in a short period may have a better positive impact on postural stability than standard physical activities, especially in a task demanding the focus of attention on other elements, where automatic processes are more responsible for postural control.

Another interesting result of our study was for the anterior-posterior range of sway variable. In the postural stability test without additional task_,_ a statistically significant main effect of intervention with a clear advantage for juggling period was observed. Despite the absence of the main effect of time, the mean differences suggested that improvements in this variable occurred only in juggling period. Period without additional juggling activity characterized by deterioration in the value of anterior-posterior range of sway. However, these results should be interpreted with caution.

Studies on experts and intermediate jugglers [65] showed similar sway amplitude in postural stability of those groups during the juggling task, but with better posture correction in favor of experts. Additionally, better juggling performance was associated with more consistent patterns of postural stability test, such as medio-lateral and anterior-posterior COP velocity or medio-lateral and anterior-posterior range of sway. Thus, better control of upper limb performance is associated with better postural control of the performer during the task. However, there are only a few evidences [65] on how mastering upper limb performance can improve general postural stability on a daily basis when people mostly maintain posture and simultaneously manipulate objects in the upper extremities. Our results showed that it is worth to look over this specific problem.

Additionally, our results showed moderate effect sizes of interaction effects for COP velocity, anterior-posterior range of sway, and anterior-posterior root mean square velocity in the postural stability test without an additional task and COP velocity and medio-lateral COP velocity in the limits of stability test in the study group but without statistical significance. All of these variables were characterized by lower values in POST juggling period. Given the sample size of our study, these results may indicate that juggling can improve postural control in healthy active older adults; however, the evidence is not conclusive.

There is a lot of evidence that physical activity can improve postural stability in older adults [22, 66]. However, better postural stability was observed after motor learning or development also among people of various ages, for example, during interventions such as gymnastics in children aged 7-11 and adults over 20 [67, 68] or during circus activities in 5- to 6-year-old children [69]. Moreover, it was observed after activities with upper limb activation as manual rhythmic movements [70] or even rifle shooting [71]. Two years of experience in circus activity training can improve postural control. The experimental group, which participated in training for two days a week, had better results of COP velocity, medio-lateral and anterior-posterior COP velocity in various conditions than the control group, which performed only recreational activities [69]. Additionally, it was proven that in the case of learning to juggle, coupling between the control of posture and manual tasks decreases. It can be understood as a better resistance to perturbations of performers during tasks, which probably translates into the manipulation of objects on a daily basis [70, 72]. Most likely, the effect of juggling on postural stability would be more pronounced for interventions among individuals without systematic physical activity.

During cognitive ability measurements, the main effect of time was observed in simple reaction time and number of correct answers in Cognitrone test, which may confirm that physical activity is somewhat effective for the maintenance and improvement of cognitive abilities. A significant amount of research confirms that a particularly moderate form of physical activity is able to improve reaction time among older adults [73, 74], especially with combined interventions of physical activity and cognitive effort [75–78]. Juggling, due to the specificity of the task and especially as a motor learning process in unexperienced people, can be interpreted as a combined form of activity [30, 34, 64, 65]. Interestingly, our results showed a medium effect size of the interaction effect of intervention and time for the variability of simple reaction time and average time of correct acceptance in Cognitrone test variables, with a clear advantage of POST juggling period. It may be speculated that this specific form of activity can have an impact on the ability to maintain attention over time. However, the results of our study did not clearly establish such an effect.

Manifestations of cognitive plasticity depend on mechanisms of neural plasticity [23]. Nevertheless, the above results indicated a much lower effect of juggling on cognitive abilities and postural stability than studies analyzing changes in brain plasticity [30, 32, 33, 39, 41–43]. Probably a higher frequency or duration of juggling intervention would increase the observed effects of cognitive abilities. However, there is a lack of studies measuring both neuronal changes and changes in cognitive abilities after a juggling intervention.

The range of physical activity of participants was lower in juggling period than in non-juggling period, but the main limitation of the CHAMPS questionnaire was that juggling could at most be considered as “other activities”. This point, however, was not taken into account when analyzing the level of physical activity of respondents. This limitation reduced the questionnaire results accordingly. Noteworthy, during the period in which respondents marked themselves with a smaller range of physical activity but experienced intervention in the form of juggling, moderate or strong effect sizes of positive changes were observed. The lack of statistical significance makes it necessary to treat these premises with caution and should be resolved in future studies.

### The strengths and limitations of the study

The strength of this study was carrying out a new form of physical activity - juggling - in the form of a training, aiming at learning a new movement on each training. The intervention was entirely tailored to the functional abilities of the older adults. In addition, this was one of the few studies that examined the effects of juggling on cognitive function and postural stability in older adults.

This study has some limitations. Despite conducting a minimum sample size analysis, it appears that the number of participants proved insufficient to clearly indicate the effects of the intervention on cognitive function and postural stability. The medium effect sizes in the absence of obtaining statistical significance seemed to confirm this limitation. Another problem may have been the number of three workouts per week. This in view of the practical aspect of the intervention was justified, but there is still a lack of certainty if with a higher frequency of juggling classes, the effects would be more noticeable for active people.

Future studies that include juggling interventions should take into account the above limitations of this study. A larger sample would have been desirable to obtain stronger results. Additionally, it may be easier to observe any statistically significant changes in samples with sedentary lifestyles or with documented disorders, in which physical activity significantly helps, than in healthy, active people. Additionally it would be valuable to compare juggling intervention not only to control non-exercising group, but also to other social activities not related to physical activities such as educational groups or film discussion session. In this case, it would be a better solution to monitor participants' physical activity throughout the whole protocol, as questionnaires can only estimate physical activity levels, whereon activity such as juggling is unclear where to be included. For studies that consider the effects of the intervention in physically active individuals, a more accurate measurement of the "physical activity" variable would be desirable, with separate consideration of non-exercise activity, exercise activity and exercises performed during the intervention. For this purpose, the use of objective methods based on accelerometry would seem appropriate. Additionally, further studies should be conducted on a larger sample of people, varying the frequency of training, and with the separation of a group of people characterized by sedentary lifestyle or with mild cognitive or postural stability impairments.

## Conclusions

Juggling in the form of a new motor task in each training unit has the potential to maintain or improve postural stability among healthy and active older adults. These changes are particularly true for medio-lateral and anterior-posterior root mean square of COP velocity variables and, to some extent, for anterior-posterior range of sway in a task requiring attention to be focused on other tasks. Moreover, the addition of juggling may induce a better effect on maintaining attention over time. A positive effect of general physical activity on cognitive functions was observed in the study, especially for reaction time and number of correct answers in Cognitrone test. Nevertheless, it can be indicated that it can be a good solution to recommend juggling, especially as lessons focused on motor learning, even for healthy and physically active older adults, expecting a positive effect. However, the clinical relevance of these changes for healthy and physically active older adults is probably low to moderate.

### Supplementary Information

Additional file 1: “Bonferroni post hoc comparison between groups for significant main or interaction effects”

## Data Availability

The datasets used and/or analysed during the current study are available from the corresponding author on reasonable request.

## Abbreviations

“PP”: main effect: time (PRE/POST)
“TR”: main effect: intervention (juggling/non-juggling)
95%CI_ES_: 95% confidence interval for effect size
95%CI_MD_: 95% confidence interval for mean difference
ANOVA RM: repeated measure analysis of variance
Area95: area 95 percentile
COG_C_: number of correct answers in the Cognitrone test
COG_CA_: average time of correct acceptations in the Cognitrone test
COG_CR_: average time of correct rejections in the Cognitrone test
COG_T_: Cognitrone test duration
COP: center of pressure
CRT: choice reaction time
DT: decision time
ES: effect size
JP: juggling period
MD: mean difference
MT: motor time
NJP: non-juggling period
*p*: p-value
POST: after period
PRE: before period
PST: postural stability test
PST_C_: standing freely without an additional task
PST_E_: standing freely with an additional task
PST_LOS_: limits of stability test
RangeX: medio-lateral range of sway
RangeY: anterior-posterior range of sway
RMS_VX_: medio-lateral root mean square of center of pressure velocity
RMS_VY_: anterior-posterior root mean square of center of pressure velocity
RT: reaction time
SD: standard deviation
SRT: simple reaction time
V_COP_: velocity of center of pressure
VCRT: variability of choice reaction time
VMT: variability of motor time
VSRT: variability of simple reaction time
VTS: Vienna Test System
ƞ_p_^2^: effect size, eta partial square
