# Supplementary material for: Effect of a juggling-based physical activity on postural stability, reaction time, and attention focus in older adults: a randomized crossover study
Source: Eur Rev Aging Phys Act. 2024 May 31;21:15. doi: 10.1186/s11556-024-00351-w (PMC11143604; doi:10.1186/s11556-024-00351-w)
Supplement: Supplementary file 1 — Additional file 1: “Bonferroni post hoc comparison between groups for significant main or interaction effects” [file 11556_2024_351_MOESM1_ESM.docx]

**Additional file 1.** Bonferroni post hoc comparison between groups for significant main or interaction effects

| Comparison between Groups  [mean] | MD | Std. Error | p-value | 95%CI_MD_ | |
| --- | --- | --- | --- | --- | --- |
| SRT: “PP” |  |  |  | **LB** | **UB** |
| PRE – POST  [305.25 \| 293.83] | 11.42 | 4.79 | **.03** | 1.55 | 21.30 |
| JP – NJP  [300.04 \| 299.04] | 1.00 | 6.12 | .87 | -11.60 | 13.60 |
| PRE JP – POST JP  [307.38 \| 292.69] | 14.69 | 5.56 | .08 | -1.24 | 23.63 |
| PRE NJP – POST NJP  [303.12 \| 294.96] | 8.15 | 5.56 | .93 | -7.78 | 24.09 |
| POST JP – POST NJP  [292.69 \| 294.96] | -2.27 | 5.56 | 1.00 | -18.21 | 13.67 |
|  |  |  |  |  |  |
| COG_C_: „PP” |  |  |  |  |  |
| PRE – POST  [54.62 \| 55.42] | -.81 | .35 | **.03** | -1.12 | -.10 |
| JP – NJP  [54.85 \| 55.19] | -.35 | .47 | .46 | -1.31 | .61 |
| PRE JP – POST JP  [54.54 \| 55.15] | -.62 | .68 | 1.00 | -2.57 | 1.34 |
| PRE NJP – POST NJP  [54.69 \| 55.69] | -1.00 | .68 | .93 | -2.96 | .96 |
| POST JP – POST NJP  [55.15 \| 55.69] | -.54 | .68 | 1.00 | -2.49 | 1.42 |
|  |  |  |  |  |  |
| RMS_VX_ in PTS_E_: „TR”x”PP” |  |  |  |  |  |
| PRE – POST  [.79 \| .96] | -.18 | .05 | **.002** | -.28 | -.07 |
| JP – NJP  [.78 \| .97] | -.19 | .05 | **<.001** | -.30 | -.09 |
| PRE JP – POST JP  [.78 \| .77] | .01 | .07 | 1.00 | -.19 | 0.20 |
| PRE NJP – POST NJP  [.79 \| 1.15] | -.36 | .07 | **<.001** | -.55 | -.17 |
| POST JP – POST NJP  [.77 \| 1.15] | -.38 | .07 | **<.001** | -.57 | -.18 |
|  |  |  |  |  |  |
| RMS_VY_ in PTS_E_: „TR”x”PP” |  |  |  |  |  |
| PRE – POST  [.97 \| 1.11] | -.14 | .06 | **.02** | -.26 | -.02 |
| JP – NJP  [1.13 \| .96] | .17 | .04 | **<.001** | .09 | 0.24 |
| PRE JP – POST JP  [1.15 \| 1.10] | .05 | .05 | 1.00 | -.10 | .21 |
| PRE NJP – POST NJP  [.79 \| 1.13] | -.34 | .05 | **<.001** | -.49 | -.18 |
| POST JP – POST NJP  [1.10 \| 1.13] | -.03 | .05 | 1.00 | -.19 | .13 |
|  |  |  |  |  |  |
| RangeY in PTS_C_: „TR” |  |  |  |  |  |
| PRE – POST  [2.68 \| 2.70] | -.02 | .11 | .84 | -.24 | .19 |
| JP – NJP  [2.59 \| 2.79] | -.19 | .09 | **.03** | -.37 | -.02 |
| PRE JP – POST JP  [2.65 \| 2.54] | .11 | .12 | 1.00 | -.25 | .46 |
| PRE NJP – POST NJP  [2.71 \| 2.86] | -.15 | .12 | 1.00 | -.51 | .21 |
| POST JP – POST NJP  [2.54 \| 2.86] | -.32 | .12 | .09 | -.68 | .03 |

SRT – simple reaction time; COG_C_ – number of correct answers; RMS_VX_ – medio-lateral root mean square velocity; RMS_VY_ – anterior-posterior root mean square of velocity; RangeY – anterior-posterior range of sway; PST_C_ – postural stability test without additional task; PTS_E_ – postural stability test with additional task (counting); “PP” – significance for main effect: time (pre/post); “TR” – significance for main effect: intervention (juggling/ non-juggling); “TR”x”PP” – significance for interaction effect of intervention and time; JP – juggling period; NJP – non-juggling period; PRE – before period; POST – after period; MD – mean difference; Std. Error – standard error; 95%CI_MD_ – 95% confidence interval for mean difference; LB – lower bound for 95%CI_MD_; UB – upper bound for 95%CI_MD_.
